# A Circulating MicroRNA Profile in a Laser-Induced Mouse Model of Choroidal Neovascularization

**DOI:** 10.3390/ijms21082689

**Published:** 2020-04-13

**Authors:** Christina Kiel, Patricia Berber, Marcus Karlstetter, Alexander Aslanidis, Tobias Strunz, Thomas Langmann, Felix Grassmann, Bernhard H.F. Weber

**Affiliations:** 1 Institute of Human Genetics, University of Regensburg, 93053 Regensburg, Germany; Christina.Kiel@klinik.uni-regensburg.de (C.K.); Patricia.Berber@klinik.uni-regensburg.de (P.B.); Tobias.Strunz@klinik.uni-regensburg.de (T.S.); Felix.Grassmann@ki.se (F.G.); 2 Laboratory for Experimental Immunology of the Eye, Department of Ophthalmology, Faculty of Medicine and University Hospital of Cologne, 50931 Cologne, Germany; marcus.karlstetter@gmail.com (M.K.); Alexander.Aslanidis@janvier-labs.com (A.A.); Thomas.Langmann@uk-koeln.de (T.L.); 3 Department of Medical Epidemiology and Biostatistics, Karolinska Institute, 171 77 Stockholm, Sweden; 4 Institute of Clinical Human Genetics, University Clinics Regensburg, 93053 Regensburg, Germany

**Keywords:** cmiRNA regulation, age-related macular degeneration, laser-induced choroidal neovascularization, biomarker

## Abstract

Choroidal neovascularization (CNV) is a pathological process in which aberrant blood vessels invade the subretinal space of the mammalian eye. It is a characteristic feature of the prevalent neovascular age-related macular degeneration (nAMD). Circulating microRNAs (cmiRNAs) are regarded as potentially valuable biomarkers for various age-related diseases, including nAMD. Here, we investigated cmiRNA expression in an established laser-induced CNV mouse model. Upon CNV induction in C57Bl/6 mice, blood-derived cmiRNAs were initially determined globally by RNA next generation sequencing, and the most strongly dysregulated cmiRNAs were independently replicated by quantitative reverse transcription PCR (RT-qPCR) in blood, retinal, and retinal pigment epithelium (RPE)/choroidal tissue. Our findings suggest that two miRNAs, mmu-mir-486a-5p and mmur-mir-92a-3p, are consistently dysregulated during CNV formation. Furthermore, in functional in vitro assays, a significant impact of mmu-mir-486a-5p and mmu-mir-92a-3p on murine microglial cell viability was observed, while mmu-mir-92a-3p also showed an impact on microglial mobility. Taken together, we report a robust dysregulation of two miRNAs in blood and RPE/choroid after laser-induced initiation of CNV lesions in mice, highlighting their potential role in pathology and eventual therapy of CNV-associated complications.

## 1. Introduction

The blood retinal barrier separates the neural retina from the circulatory system. It is comprised of an inner barrier, formed by tight junctions between adjacent retinal endothelial cells, and an outer network of tight junctions between adjacent retinal pigment epithelium (RPE) cells. The two barriers constitute a sophisticated system to allow the diffusion of very small molecules such as oxygen and glucose to the neural retina, while inhibiting the passive flux of larger particles. Pathological processes, such as choroidal neovascularization (CNV), disrupt this barrier with detrimental consequences for retinal homeostasis. Prior to CNV, the retina responds to hypoxic stress by upregulating the expression of vascular endothelial growth factor (VEGF), which in turn results in the ingrowth of new blood vessels from the choriocapillaris [1,2,3]. Such fragile vessels invade the RPE monolayer, leaking serous fluid, lipids, and blood into the neural retina. Consequently, CNV is associated with a number of pathological conditions, including myopia and osteogenesis imperfecta, but most commonly occurs in the neovascular late-stage form of age-related macular degeneration (nAMD) [4,5,6].

AMD is a complex disease of the central retina, and is the third leading cause of blindness worldwide owing to atrophic or neovascular manifestations [7]. In nAMD, the formation of CNV lesions is initially accompanied by an inflammatory response, which eventually results in the formation of scar tissue [8,9]. While in early AMD, the initial decrease in visual acuity is slow and often remains unnoticed, the onset of CNV is consistently associated with rapid vision loss [10]. Understanding the etiology of AMD and its late complications has challenged retinal researchers for decades, while in more recent years, it is commonly agreed upon that genetic predisposition and environmental influences are the main risk factors driving AMD pathology [11,12,13]. Recently, evidence has emerged that suggests that epigenetic mechanisms, such as microRNA (miRNA) modulated gene expression, are likely to be involved in early disease processes [14,15]. MiRNAs are short, non-coding RNAs, which bind to and degrade mRNA transcripts, thereby modifying gene expression. Although initially thought to be confined to intracellular modes of action, miRNAs were demonstrated by Chim and colleagues to be present extracellularly as so-called circulating miRNAs (cmiRNAs) in blood plasma [16]. Since then, cmiRNAs have been recognized as an exciting new avenue of research for many pathologies, mainly owing to their potential in diagnostic and therapeutic applications. Multiple groups have previously investigated differentially expressed cmiRNA in nAMD patients, but so far, there is no consensus about which cmiRNAs are truly dysregulated [17].

The aim of this study was first to provide clarity in the field of cmiRNA dysregulation in nAMD patients, by reviewing and summarizing previous publications. Further, we intend to identify robustly dysregulated cmiRNAs in a laser-induced CNV mouse model. This model involves the use of controlled argon laser irradiation to locally puncture Bruch’s membrane [18,19]. After treatment, the mice develop a characteristic- and time-dependent inflammatory and angiogenic ocular response [18]. Initially, the mice develop an immunological reaction during which time inflammatory cells such as neutrophils and macrophages invade the choroid and resident retinal microglia are activated [20]. In contrast to this early reaction, the onset of angiogenesis peaks around day 7, but is still relatively prominent at day 14 after treatment [18].

Here, we analyzed global alterations in murine cmiRNA expression at various time points after laser treatment. The most strongly dysregulated cmiRNAs were then independently quantified by real-time reverse transcription PCR (RT-qPCR) in blood, retinal, and RPE/choroid tissue. We identified two reproducibly dysregulated cmiRNAs and performed a series of in vitro assays in murine microglial and endothelial cells, characterizing functional effects of these miRNAs on cell viability, migration, vascularization, and immune regulation.

## 2. Results

### 2.1. Literature Search for cmiRNA Dysregulation in nAMD Patients

**Figure 1:**
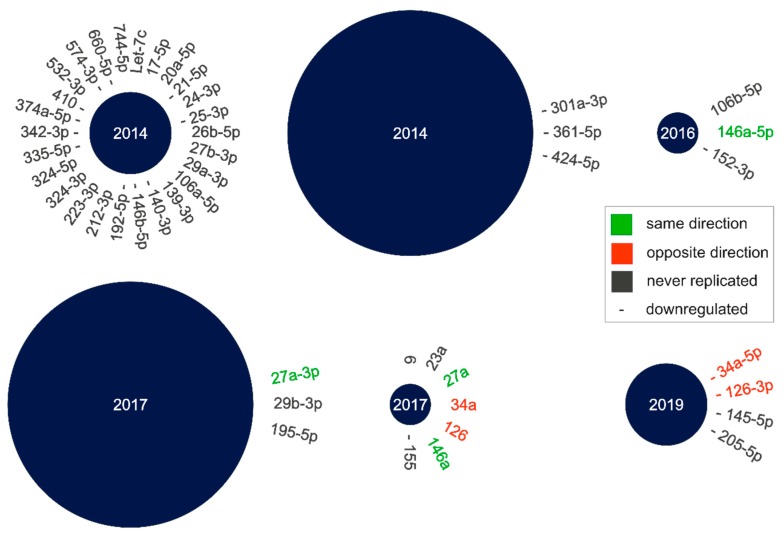
Circulating microRNA (cmiRNA) dysregulation in neovascular age-related macular degeneration (nAMD) patients summarized from literature data. Each circle represents one publication, which investigated cmiRNAs in human nAMD patients. The size of each circle represents the number of participants included in the study (small circle: <49 participants, medium circle: 50–149 participants, large circle: >150 participants). The year the study was published is denoted inside each circle. The cmiRNAs identified by each study are listed around the circle, and downregulated cmiRNAs are marked with a dash. The prefix of each cmiRNA is hsa-mir-, except for hsa-let-7c. Two cmiRNAs were upregulated in patients in two studies, hsa-mir-146a-5p and hsa-mir-27a-3p (highlighted in green). In contrast, two other cmiRNAs were upregulated in one study, and downregulated in another, hsa-mir-34a and hsa-mir-126 (highlighted in red). Of note, one publication did not indicate which mature strand (3p or 5p) was investigated. In total, 38 of the 42 (90%) cmiRNAs differentially regulated in nAMD patients have not been replicated in independent studies.

So far, several studies have investigated cmiRNA dysregulation in nAMD patients, but they exhibit a high variability of the identified cmiRNAs. To enable a comprehensive overview of dysregulated cmiRNAs in nAMD, we performed a literature search, and compiled the dysregulated cmiRNAs that were identified by each study (Figure 1; Supplementary Table S1). Only studies that investigated cmiRNAs in serum, plasma, or whole blood of nAMD patients were included. Six studies fulfill these criteria, and they identified in total 42 different cmiRNAs to be related to nAMD [21,22,23,24,25,26]. Only two cmiRNAs were identified in two independent studies, which were both upregulated (hsa-mir-146a and hsa-mir-27a) [23,24,25]. In contrast, two additional cmiRNAs were also identified in two independent studies, but in opposite directions. One study showed that hsa-mir-34a and hsa-mir-126 were upregulated in their patient cohort, while another study showed that these cmiRNAs were downregulated in their patient cohort [24,26].

Of special note, a single study detected all four of the previously mentioned cmiRNAs [25]. However, the results of this study should be regarded with caution, as they may not adequately identify the mature miRNA they investigated. An important step in miRNA biogenesis is the processing of the pre-miRNA hairpin structure to a mature miRNA duplex [27,28,29]. This duplex results in two functional miRNA strands, and the directionality of these strands is used to differentiate between them. The 5′ end of the hairpin results in the 5p strand, while the 3′ end results in the 3p strand. The proportion between the functional 5p and 3p strands varies. In some cases, both strands are functional, but it can also be skewed towards one strand or the other [30,31]. Although most publications that investigated cmiRNA expression in nAMD patients documented the strand they detected, this one publication mentioned above did not [25]. Consequently, one cannot be certain about the true nature of the respective cmiRNAs in this study.

Importantly, the vast majority of cmiRNAs (38/42) differentially expressed in nAMD patients in one study have never been replicated in an independent study. Although some variability is to be expected, the inconstancy of these results prohibits the identification of distinct cmiRNAs that show a robust differential expression in nAMD patients.

### 2.2. CmiRNA Expression after Laser-Induced CNV Formation in C57Bl/6 Mice

**Figure 2:**
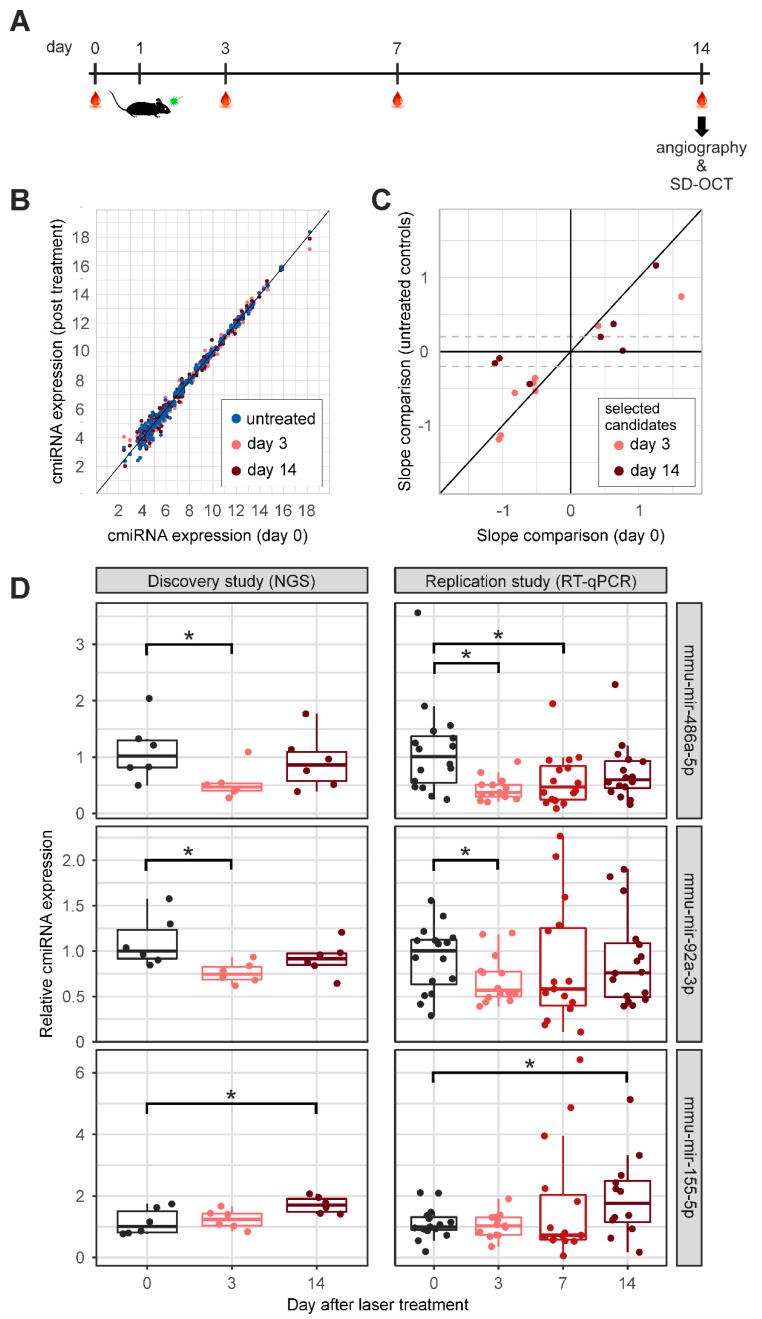
CmiRNA regulation after laser-induced choroidal neovascularization (CNV) in a discovery and a replication study. (**A**) Mice received laser treatment on day 1, and blood was drawn on day 0 (baseline reference), days 3 and 7 (only in the replication study), and on day 14. On day 14, CNV formation was confirmed via angiography and SD-OCT. (**B**) Comparison of cmiRNA profiles of untreated, day 3, or day 14 samples with day 0 samples. This analysis demonstrates an overall stable cmiRNA expression after laser treatment. (**C**) On the *x*-axis, the slopes of candidate cmiRNA expression on day 0 compared to day 3 or day 14 are shown. The slope of these cmiRNAs are given on the y-axis, when analyzed in comparison with untreated control mice. The dotted lines represent the slope threshold of an absolute slope > 0.2. Candidates in between the dotted lines were excluded from further analysis. (**D**) The relative expression values of three significantly dysregulated cmiRNAs in the discovery study and replication study are shown. For the independent replication study using quantitative reverse transcription PCR (RT-qPCR), two additional batches of mice were treated with an argon laser, including 6 and 12 mice, respectively. Shown are relative expression values normalized to the median expression on day 0 for the three cmiRNAs. All three cmiRNAs were differentially regulated at one or several time points of measurement (day 3, 7, and 14) in comparison with day 0. * *p*-value < 0.05 (linear regression model). SD-OCT, spectral domain optical coherence tomography; NGS, next generation sequencing.

CmiRNAs were determined in mice after laser-induced CNV formation in a discovery and an independent replication study. Initially, six mice were treated with an argon laser to induce CNV on day 1. Blood samples were drawn on days 0 (baseline), 3, and 14 (Figure 2A). On day 14, CNV was confirmed via angiography and spectral domain optical coherence tomography (SD-OCT). In the discovery study, cmiRNA expression profiles were analyzed via global RNA sequencing 3 and 14 days after laser treatment, respectively. CmiRNA expression profiles from day 0 (one day prior to laser treatment) and 5 untreated mice (age-matched with day 14 samples) were used as controls. The cmiRNA expression profiles were compared between all groups, demonstrating that the expression of most cmiRNAs remained stable upon laser treatment (Figure 2B; Supplementary Table S2). To detect cmiRNAs dysregulated after laser treatment, we initially compared cmiRNA expression on day 0 (baseline) with day 3 and day 14 after treatment, as well as day 3 and day 14 with each other. By placing thresholds at *p*-value < 0.05 and absolute slope > 0.4, cmiRNAs were selected from each group for further analysis (Supplementary Table S3). The expression of the selected cmiRNAs was then correlated in a second comparison with the data from the untreated control animals. Here, we assumed that cmiRNA expressions at day 0 baseline and untreated control mice are comparable. To this end, we tested if any of the potential candidates revealed an altered expression between these two control groups, and applied the same criteria as in the initial comparison (*p*-value < 0.05 and absolute slope > 0.4). This excluded cmiRNA mmu-mir-326-3p from further analysis (Supplementary Table S4). Then, the expression of the remaining candidates in the untreated control mice was analyzed in comparison with the expression at day 3 or day 14. Only cmiRNAs that replicated the slope direction and an absolute slope > 0.2 were considered for the replication study (Figure 2C, Supplementary Table S4).

Nine remaining cmiRNAs were subjected to an independent replication by RT-qPCR in two independent batches of animals, including 6 and 12 mice treated with an argon laser on day 1, and a blood withdrawal scheme on days 0, 3, 7, and 14 (Figure 2A). Note that the data set in the replication study was enriched for samples from day 7, to more accurately measure cmiRNA expression over time. Seven of the cmiRNAs showed an expression slope in the same direction as in the discovery study (Supplementary Table S5). Three cmiRNAs, mmu-mir-486a-5p, mmu-mir-92a-3p, and mmu-mir-155-5p, were significantly regulated at one or several time points after laser treatment (*p*-value < 0.05, Figure 2D, Supplementary Figure S1).

### 2.3. MiRNA Expression in Retinal and RPE/Choroidal Tissue

**Figure 3:**
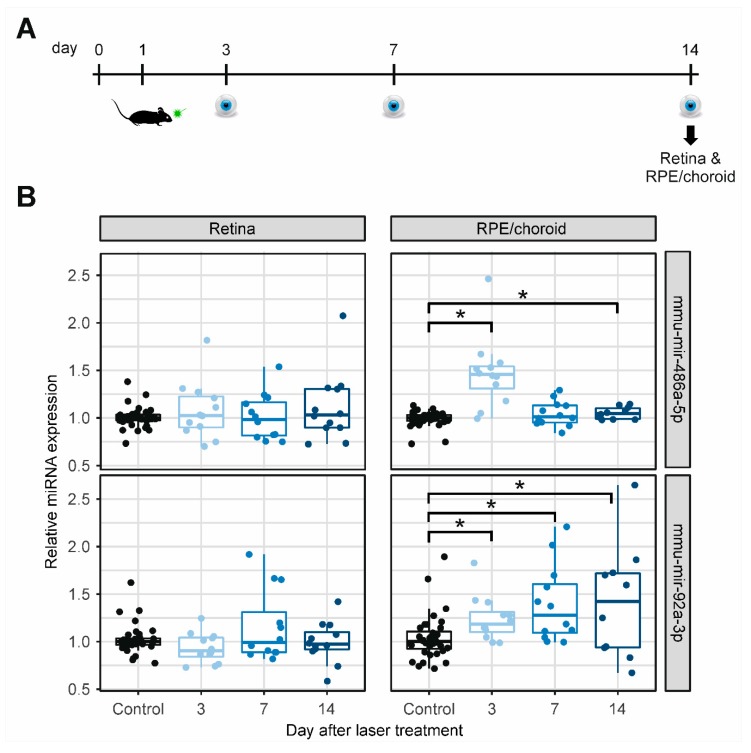
MiRNA regulation in ocular tissue after laser-induced CNV. (**A**) Mice were treated with an argon laser on day 1, and retinal and retinal pigment epithelium (RPE)/choroidal tissue samples were isolated from 12 mice per time point on days 3, 7, and 14, as well as from untreated control mice. (**B**) Relative miRNA expression levels of the two cmiRNAs dysregulated in blood samples, retinal tissue, and RPE/choroidal tissue. Given are relative expression levels normalized to the median of untreated control mice. * Q-value < 0.05 (linear regression model, false discovery rate (FDR) corrected).

The expression of two significantly and robustly dysregulated cmiRNAs (mmu-mir-486a-5p and mmu-mir-92a-3p) was further investigated in ocular tissues by RT-qPCR. Mmu-mir-155-5p showed strong expression fluctuations and a small effect size (slope < 0.06) in blood and was not further investigated. For this analysis, 36 mice were treated with argon laser on day 1, and retinal and RPE/choroidal tissues were extracted from 12 mice, on day 3, day 7, and day 14 (Figure 3A), respectively. MiRNA expression was compared to those from the 12 untreated control mice. In retinal tissue, neither mmu-mir-486a-5p nor mmu-mir-92a-3p was significantly dysregulated after laser treatment at any time point (Figure 3B, Supplementary Figure S2A, Supplementary Table S6). In the RPE/choroidal tissue, the two miRNAs showed a statistically significant upregulation after laser treatment for at least two time points each (Q-value < 0.05, Figure 3B, Supplementary Figure S2B, Supplementary Table S6). For mmu-mir-486a-5p, the strongest dysregulation was observed on day 3 (slope = 0.408 (0.188–0.628)). In contrast, the expression of mmu-mir-92a-3p increased steadily over time (slope on day 14 = 0.466 (0.084–0.848)).

### 2.4. Functional Characterization of miRNAs in Murine Endothelial and Microglial Cells

**Figure 4:**
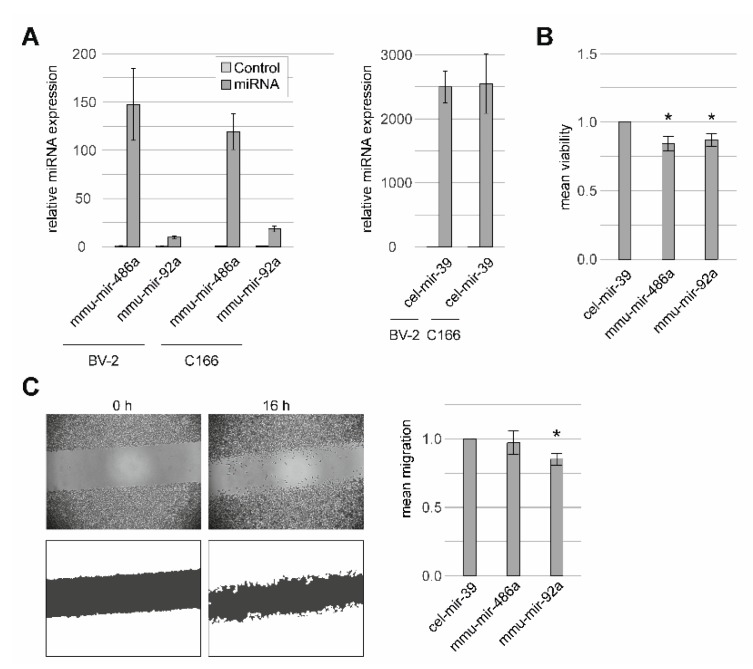
Functional characterization of miRNAs in murine microglial cells (BV-2) and murine endothelial cells (C166). (**A**) Successful overexpression of miRNAs in both cell lines was verified via RT-qPCR 48 h after transfection. Overexpression was normalized to endogenous expression of the respective miRNA. Three independent experiments were performed, each with 2-3 technical replicates. Bars indicate standard errors (SEs). (**B**) Effect of miRNA overexpression on BV-2 cell viability, which was normalized to the viability of transfected cells with control miRNA cel-mir-39-3p. Seven independent experiments were performed, with 6-8 technical replicates each. Bars indicate SEs. (**C**) Effect of miRNA overexpression on the migration of BV-2 cells. A scratch assay was performed, and cell migration was defined as the percentage area closed after 16 h incubation. Seven independent experiments were performed, with 4-6 technical replicates each. Bars indicate SEs. * Q-value < 0.05 (Kruskal-Wallis test, followed by a Dunn’s multiple comparison test to receive uncorrected *p*-values for the comparisons of interest, which were corrected using FDR).

A series of experiments were performed to investigate the functional effects of the two candidate miRNAs in murine microglial (BV-2) and murine endothelial cells (C166). By RT-qPCR, we initially verified that all miRNAs were overexpressed successfully in the two model cell lines (Figure 4a, Supplementary Table S7). Although miRNAs mmu-mir-92a-3p and mmu-mir-486a-5p were overexpressed, we observed fluctuations in the expression profiles, ranging from 9.7-fold for mmu-mir-92a-3p in BV-2 to 147.6-fold for mmu-mir-486a-5p also in BV-2. These effects may be the result of a variable endogenous expression of the respective miRNAs. Of note, the control miRNA cel-mir-39-3p (which has no homologue in the mouse) showed the strongest overexpression of around 2500-fold in both cell lines used (Figure 4A).

Overexpression of mmu-mir-92a-3p and mmu-mir-486a-5p in the murine microglial cell line BV-2 resulted in a significant reduction of cell viability, in comparison with cells transfected with control miRNA cel-mir-39-3p (Figure 4B, Supplementary Table S8). Furthermore, murine microglial cells transfected with mmu-mir-92a-3p also showed a reduced migration in a scratch assay (Figure 4C, Supplementary Table S8). To measure the response of murine microglial cells to immunological stimuli, cells were stressed with lipopolysaccharide (LPS) and nitric oxide (NO) levels were quantified [32]. However, no significant response of miRNA overexpression in murine microglia was observed upon LPS stress (Supplementary Table S8). We also did not observe a significant effect of miRNA overexpression in murine endothelial cells C166 on network length in a tube formation assay, on cell migration in a scratch assay, or on cell viability (Supplementary Table S8).

## 3. Discussion

Laser induction of CNV lesions in the mouse eye is a commonly used in vivo model to induce inflammation and angiogenesis—two hallmarks of nAMD [18,19]. As far as we could find out, this model has never been used in the context of miRNA regulation. We used laser treatment to induce the formation of CNV lesions in mice, and identified three cmiRNAs that were reproducibly dysregulated. We further demonstrated that two robustly dysregulated cmiRNAs also display an altered expression in RPE/choroidal tissue after laser treatment. Interestingly, the observed effects for both miRNAs in ocular tissue are in the opposite direction when compared with blood.

Several studies have investigated serum and plasma cmiRNA levels in nAMD patients [21,22,23,24,25,26], but the findings in-between studies appear difficult to reproduce [17]. This emphasizes the need for alternative approaches to detect robustly dysregulated cmiRNAs in CNV. While miRNAs are known to maintain cellular homeostasis by buffering against cellular and organismal stress [33], many factors seem to influence fluctuations in cmiRNA expression. We designed our experiments to minimize these effects, and aimed to identify cmiRNAs that were dysregulated solely owing to the laser treatment. We analyzed cmiRNA expression profiles in several steps, with less strict *p*-value thresholds initially to focus on the reproducibility of expression trends. Therefore, candidate cmiRNAs had to pass several stages of analysis, including comparisons with two control groups (day 0 before treatment and untreated mice), and an independent replication study with two independent batches of animals. We did not perform correction for multiple testing at these early steps of analysis, instead, candidate cmiRNAs had to show consistent effect sizes and pass several *p*-value thresholds. Consequently, this approach identified three highly valid and reproducibly dysregulated cmiRNAs, namely, mmu-mir-486a-5p, mmu-mir-92a-3p, and mmu-mir-155-5p.

An interesting cmiRNA detected in our analysis initially, but not followed through the study is mmu-mir-155-5p, which was upregulated in blood on day 14 with a 1.8-fold increase. This is one of the strongest expression differences we observed in our analysis, compared with the other two significantly differentially expressed cmiRNAs (strongest fold change of mmu-mir-486a-5p = 0.62, mmu-mir-92a-3p = 0.85). In contrast, the regression model showed a rather moderate effect size (slope) for mmu-mir-155-5p, in comparison with the other cmiRNAs. By definition, “fold change” refers to differences between the mean expression values of two groups. In vivo models are complex, and include many sources of variability and confounding effects. We thus adjusted for known confounders by applying regression models, and chose to set thresholds using the output of the regression model (slope and *p*-value). Besides this statistical issue, differential expression of mmu-mir-155-5p was reported for several diseases, including immune response to infection [34,35], Alzheimer’s disease [36], multiple sclerosis [37], and cancer [38,39,40]. On the basis of the fluctuation of values we observed, and the known associations with other diseases, we questioned the benefit of mir-155-5p as a specific and reliable biomarker for nAMD.

Mmu-mir-92a-3p and mmu-miR-486a-5p were significantly dysregulated in our study. In blood, both cmiRNAs were downregulated on day 3, and in RPE/choroid, they were upregulated inter alia on day 3. Thus, in our study, these two miRNAs are regulated in different directions in different tissues, although at the same time point since setting the retinal insult. These results corroborate previous findings, which support dynamic differences in intercellular miRNA expression profiles. A recent study investigated cell-type specific patterns of miRNA expression, and revealed profound differences, even in closely related cell types [41]. Furthermore, another study that co-sequenced single cell miRNA and mRNA theorized that intercellular mRNA heterogenity could be caused by miRNA heteregenity, and thereby help to explain non-genetic cell to cell variability [42]. Even more interesting is the concept that the function of miRNA can vary based on the cell type. In a previous study, overexpression of mir-92a resulted in increased proliferation in a breast cancer cell line and an acute promyeloic leukemia cell line, but increased apoptosis in a T cell leukemia cell line [43]. The differences in miRNA expression as defined in our study may not be artificial findings, but instead could reflect functional characteristics and so far only little is understood in the context of neovascular injury of the retina.

In our analysis, mmu-mir-486a-5p and mmu-mir-92a-3p were downregulated in blood at day 3 after treatment, which is characterized by an immune reaction owing to laser treatment [18,20]. At this time point, neutrophils and macrophages invade the choroid and resident retinal microglia are activated [20]. To investigate the effect of these two miRNAs in vitro, we overexpressed each miRNA in murine microglial cells, known as the immunological watchdogs of the retina [44]. We observed a reduced viability of microglial cells for each miRNA, and a reduced microglial migration rate for mmu-mir-92a-3p. We theorize that downregulation of mmu-mir-486a-5p and mmu-mir-92a-3p leads to an enhanced microglia function, and thus could be correlated to the observed immune reaction at this time point. An involvement of miRNAs in the immune response is well documented [45,46]. Specifically, a landmark study showed that mir-92a was involved in cell–cell communication of T and B lymphocytes [47], key mediators of the bodies’ humoral immune response. Overall, we postulate that expression changes observed in blood are part of the murine immune reaction after laser treatment.

In contrast to blood, mmu-mir-486a-5p and mmu-mir-92a-3p were upregulated in RPE/choroid after laser treatment. A previous study demonstrated that, in the human RPE cell line ARPE-19, the human homologue to mmu-mir-486a-5p, hsa-mir-486-5p, was upregulated upon oxidative stress [48]. This is an interesting finding because oxidative stress is known to be critical in AMD pathogenesis [49]. Similarly, hsa-mir-92a was upregulated in human umbilical vein endothelial cells after oxidative stress induction. In our study, both miRNAs were upregulated in RPE and choroidal tissue, whose main components are RPE and endothelial cells. Possibly, upregulation of mmu-mir-486a-5p and mmu-mir-92a-3p is a cell-type specific effect of RPE and endothelial cells upon a laser-induced stress reaction, comparable to oxidative stress.

Interestingly, upregulation of mmu-mir-92a-3p in RPE/choroidal tissue increased over time. In the laser-induced CNV model, an angiogenic reaction follows the immune reaction. The neovascular surface area peaks at day 7, and is still relatively prominent at day 14 [18]. The increased expression of mmu-mir-92a-3p in RPE/choroidal tissue during these time points could indicate an involvement of this miRNA in the ocular angiogenic reaction. Previously, the impact of this miRNA on angiogenesis was studied, although with contradicting results [50,51,52,53]. So far, there is no consensus on whether mir-92a is a positive or negative regulator of angiogenesis. It was argued that the inconsistent results are owing to small variations in the experimental set-up [51]. This could explain why we failed to identify an effect of mmu-mir-92a-3p in the tube formation assay in murine endothelial cells. Still, mmu-mir-92a-3p may have a consequence on angiogenesis in our in vivo model, even though there was no recordable effect in the in vitro assay.

Taken together, we found a robust differential regulation of three cmiRNAs in blood of laser-treated mice established to represent a model of nAMD. Two of these cmiRNAs, mmu-mir-486a-5p and mmu-mir-92a-3p, were followed up in further experiments. Both were downregulated in blood and upregulated in RPE/choroidal tissue after laser treatment, which could indicate cell-type specific regulation and function. In blood, both miRNAs are likely involved in an immune reaction, while in RPE/choroidal tissue, a stress reaction may be comparable to an oxidative stress reaction.

## 4. Materials and Methods

### 4.1. Ethical Statement on Animal Studies

This animal study conformed to the Association for Research in Vision and Ophthalmology (ARVO) Statement for the Use of Animals in Ophthalmic and Vision Research, and was approved by the government of North Rhine-Westphalia (ID: 84-02.04.2015.A413).

### 4.2. Literture Analysis of Differentially Expressed cmiRNAs in nAMD Patients

A literature search was performed in Pubmed in March 2020. Studies that investigated cmiRNA dysregulation in nAMD patients were compiled. In total, six studies met these criteria [21,22,23,24,25,26]. Studies that investigated intracellular cmiRNAs [54,55], cmiRNAs in exosomes [56], a combination of nAMD and geographic atrophy AMD patients [57,58], and a combination of nAMD and congenital hemochromatosis patients [59] were excluded.

### 4.3. Induction of CNV Lesions in the Mouse Model

CNV was induced in mice as previously described, with minor modifications [60]. In this study, female drug and test naїve C57Bl/6J mice were used. Mice were 78 days old, with the exception of the tissue studies, which were 56 days old. Mice from the tissue studies’ harvest on day 14 also served as second batch for the blood replication study, and were 56 days old. The mice were anesthetized with Rompun (6 mg/kg) and Ketavet (100 mg/kg), and pupils were dilated with drops of Phenylephrine HCl (0.25%)–Tropicamide (0.05%). For the laser procedure, 2% Methocel (OmniVision, Puchheim, Germany) was applied topically before adding a microscopy cover slip to enable precise laser induction. The fundus was visualized with an imaging camera, and laser photocoagulation was induced using the image-guided laser system. Six laser burns at an equal distance from the optic nerve were induced in each eye by a green Argon laser pulse with a wavelength of 532 nm, a fixed diameter of 50 μm, duration of 100 ms, and power levels of 150 mW. Eyes that developed vitreous bleeding, cataract, or keratopathy were excluded from the analysis.

Mice were then placed on a pre-warmed warming plate at 35 °C, until they regained consciousness. SD-OCT was performed using the image-guided OCT system as previously described [17].

### 4.4. Blood and Tissue Sample Preparation and cmiRNA Isolation

For the discovery study, six mice were treated with an argon laser on day 1, and blood was drawn on day 0 (one day before treatment), 3, and 14. Blood was drawn once from five untreated mice age-matched to day 14 laser-treated samples. For the replication study, two independent batches of mice were treated with argon laser, and blood was drawn on day 0, 3, 7, and 14. The first batch consists of six mice and the second batch of 12 mice. Retinal and RPE/choroidal tissue from day 14 was taken from the same mice that were used for blood collection in batch 2. Tissue was extracted from 12 additional laser treated mice per time point on day 3 and 7. Ocular miRNA expression in laser treated mice was compared to the expression in the 12 untreated mice.

For the blood samples, ~40 µL of peripheral venous blood was drawn via the facial vein and mixed with 120 µL PAXgene Blood RNA tube stabilizing solution (PreAnalytiX GmbH, Hombrechtikon, Switzerland). Prior to RNA isolation, samples were centrifuged for 10 min at 2800× *g* at 4 °C, and the supernatant (~150 μL) was collected and used for RNA isolation. For the tissue samples, the retina and RPE/choroid was extracted and snap frozen in liquid nitrogen. Prior to RNA isolation, tissue samples were homogenized in 300 μL lysis buffer with pestles, and further disintegrated by passing them through 18 G, 20 G, and 23 G needles. RNA isolation from blood and tissue was performed according to the procedures for organic extraction and total RNA isolation from the mirVANA microRNA isolation kit (Ambion, Waltham, MA, USA) as previously described [22,61], with the following modifications. The centrifugation step during the phenol/chloroform extraction was performed for 30 min at 4 °C. Finally, RNA was eluted in 50 μL of nuclease-free water (Ambion, Waltham, MA, USA).

### 4.5. Sequencing of cmiRNAs and Data Analysis (Discovery Study)

cDNA libraries were constructed from blood samples using the NEXTflex™ Small RNA-Seq Kit v3 (Bioo Scientific, Austin, TX, USA), according to the manufacturer’s instructions. The cDNA libraries were multiplexed using the multiplex primers included in the kit. The resulting cDNA libraries were purified using AMPure beads (Beckman Coulter, Brea, CA, USA), and their concentration and size distribution were determined on an Agilent BioAnalyzer DNA high-sensitivity Chip (Agilent Technologies, Santa Clara, CA, USA). Up to 12 libraries were pooled at equimolar concentrations. Sequencing was performed on a MiSeq Desktop Sequencer using the MiSeq Reagent Kit v3 with 150 cycles (Illumina, San Diego, CA, USA). As most of the reads are expected to be from very few cmiRNAs (similar to human studies [22]), we used a conservative 20% PhiX spike-in, to avoid sequencing problems associated with low diversity libraries.

The obtained sequencing data were analyzed with the mirDEEP2 package [62]. Briefly, all reads were mapped to the murine genome (mm10) using BowTie2 [63]. Reads that failed to align were excluded. Remaining reads were mapped to the pre-miRNA and miRNA sequences obtained from mirbase.org (Release 21 June 2014) and quantified. Using the statistical software R [64], the data were normalized using a trimmed mean of M-values (TMM) algorithm implemented in the edgeR package [65], and transformed with the binary logarithm. In order to account for the presence of batches in the data occurring through independent sequencing runs, we employed an empirical Bayesian batch effect correction algorithm known as ComBat [66].

For the analysis of cmiRNAs that were assayed over several time points, we used linear mixed effects model implemented in the nlme package [67]. CmiRNAs were considered to be potential candidates if the *p*-value was below 0.05 and the absolute slope was above 0.4. For the comparison of the two control groups (day 0 before laser treatment, and untreated control mice), a Firth’s bias-reduced logistic regression model implemented in the logistf package [68] was used. CmiRNAs with a *p*-value below 0.05 and an absolute slope above 0.4 in the comparison of day 0 mice with untreated mice were excluded from further analysis. Candidates were validated in a comparison of treated and untreated control mice using linear fixed effects models implemented in R. Candidates were considered to be validated if the slope indicated the same direction as in the comparison with day 0 control group, and only candidates with an absolute slope above 0.2 were retained.

### 4.6. RT-qPCR and Data Analysis (cmiRNA Replication Study and miRNA Expression in Tissue)

MiRNA reverse transcription followed by RT-qPCR was performed according to Hurteau et al. [69]. Briefly, 50 ng of purified cmiRNA solution were modified by *E. coli* Poly (A) Polymerase I (E-PAP) by the addition of a polyA tail (Ambion, Waltham, MA, USA). Reverse transcription was performed with Superscript III reverse transcriptase (Invitrogen, Waltham, MA, USA) and a Universal RT oligonucleotide primer, which contains a polyT stretch of DNA that binds to the newly synthesized polyA tail (Supplementary Table S8). The RT solution was diluted 1:40, except for samples that had previously been in long-term storage, which were diluted 1:10. Then, 4 µl diluted RT solution was used per RT-qPCR reaction. Each RT-qPCR master mix was prepared according to the protocol of the Power SYBR Green Master Mix (Applied Biosystems, Waltham, MA USA or Eurogentec, Cologne, Germany), and run on an ABI Viia-7 (Applied Biosystems, Waltham, MA, USA) or a QuantStudio 5 (Applied Biosystems, Waltham, MA, USA). Each miRNA was assayed in triplicates (primer Sequences are listed in Table S8). Primers that performed poorly (<50% RT-qPCR efficiency) were excluded from further analysis. Primers for mature miRNA detection in retinal and RPE/choroidal tissue as well as long-term stored blood samples were modified as reported elsewhere [70] (oligonucleotide primer sequences are listed in Supplementary Table S9). We further excluded measurements with a standard deviation greater than 0.4 Ct values in the triplicates. We used the TMM normalization algorithm implemented in the edgeR package [65], in order to normalize the Ct-values according to the amount of isolated RNA and reverse transcription efficiency. CmiRNA expression in blood was normalized to the median of day 0 expression, and miRNA expression in tissues was normalized to the median of untreated controls, to reduce potential batch effects. All batches were normalized separately.

For the RT-qPCR analysis of cmiRNAs, we used the linear mixed effects model implemented in the nlme package [67]. Candidates were considered to be replicated if the slope was in the same direction as in the discovery study, and the observed *p*-value was below 0.05. For the analysis of miRNAs in retinal and RPE/choroidal tissue, we used a linear fixed effects models implemented in R. *p*-values were adjusted per day and tissue, according to the FDR [71], implemented in the multtest package [72]. FDR values (Q-values) below 0.05 were considered significant.

### 4.7. Cell Culture and miRNA Mimic Transfection

C166 cells (murine endothelial yolk sac cells) (American Type Culture Collection, ATCC, Manassas, VA, USA) were cultivated in Dulbecco’s Modified Eagle Medium (DMEM) high glucose medium containing 10% FCS (fetal calf serum). BV-2 cells (murine microglial cells) were grown in DMEM high glucose medium containing 5% FCS and 195 µM β-Mercaptoethanol. Cells were transfected with miRCURY LNA miRNA mimics (Qiagen, Hilden, Germany), and cel-mir-39-3p was used as negative control (sequences are given in Supplementary Table S10). BV-2 cells were transfected with Lipofectamine RNAiMax (Thermo Fisher Scientific, Waltham, MA, USA), according to the manufacturer’s protocol. C166 were transfected with HiPerFect (Qiagen, Hilden, Germany), according to the manufacturer’s protocol. Functional assays were performed 48 h after transfection.

### 4.8. RT-qPCR to Detect Overexpression

For quantification of miRNA overexpression, cells were seeded on a six well plate. RNA was isolated 48 h after transfection, with the mirVANA microRNA isolation kit (Ambion, Waltham, MA, USA), as described previously, with minor modifications. Cells were harvested by adding 300 µL of lysis buffer and cells were detached using cell scrapers. RT-qPCR was performed as described above. Ct values greater than 0.4 were excluded, and overexpression was normalized to cel-miR-39-3p transfected cells as control.

### 4.9. Tube Formation Assay

The tube formation assay was performed with the murine endothelial cell line C166 as described elsewhere [73] with some modifications. Extracellular matrix Geltrex LDEV-Free Reduced Growth Factor Basement Membrane Matrix (Thermo Fisher Scientific, Waltham, MA, USA) was used to coat 96-well plates, with 37.5 µL per well. Plates were centrifuged 30 min at room temperature at 800–1000 rpm, and incubated afterwards for at least 15 min at 37 °C to allow gelling of the Geltrex. Before seeding the C166 cells on 96-well plates, the plates were washed with 50 µL of DPBS (Dulbecco’s phosphate buffered saline, Sigma Aldrich, St. Louis, MO, USA) per well. C166 cells were detached by adding trypsin-ethylenediaminetetraacetic acid (EDTA) solution, and trypsin was stopped by adding 20% FCS in DPBS. Cells were centrifuged for 7 min at 1200 rpm, and the pellet was resuspended in 1/3 EGMPlus Endothelial Cell Growth Media-Plus with EGMPlus SingleQuots supplements (Lonza, Basel, Switzerland) and 2/3 EGMPlus Endothelial Cell Growth Media-Plus without SingleQuots supplements. Cells were counted with a CASY Cell Counter (OLS OMNI Life Science, Bremen, Germany) and 10,000 cells per well were seeded on the coated 96-well plate. Photos were taken after 5 h incubation at 37 °C, and the total length as well as the total segments length of the cellular network was determined using the Angiogenesis Analyzer [74] for ImageJ.

### 4.10. Scratch Assay

Scratch assays were performed as described elsewhere [75] with some modifications. Twenty-four hours after transfection, cells were seeded on 96-well plates. C166 cells were seeded on uncoated plates, while BV-2 cells were seeded on plates coated with Poly-L-lysine hydrobromide (Sigma Aldrich, St. Louis, MO, USA). After 24 h incubation at 37 °C, scratches were introduced into the confluent monolayer of cells, using a WoundMaker (Essen BioScience, Ann Arbor, MI, USA). Photos were taken 0 h and 16 h after scratch induction. During this time, C166 cells were incubated at 37 °C with normal cultivation medium and BV-2 cells were incubated with a reduced medium (DMEM high glucose medium containing 1% FCS and 286 µM β-Mercaptoethanol (Sigma Aldrich, St. Louis, MO, USA)). Scratch areas were determined using ImageJ, and migration ability was defined as the percentage area closed after 16 h incubation.

### 4.11. Viability

Twenty-four hours after transfection, cells were seeded on 96-well plates and incubated for 24 h. Cell viability was measured using the Cell Counting Kit - 8 (Sigma Aldrich, St. Louis, MO, USA), according to the manufacturer’s protocol. Absorbance at 455 nm was measured after 2 h incubation, using a Spark microplate reader (Tecan, Männedorf, Switzerland).

### 4.12. NO Assay

Twenty-four hours after transfection, BV-2 cells were stressed with 200 ng/mL LPS from Escherichia coli (Sigma Aldrich, St. Louis, MO, USA). After 20 h incubation at 37 °C, the NO concentration in the supernatant was measured using the Griess Reagent System (Promega, Madison, WI, USA), according to the manufacturer’s protocol. Absorbance at 540 nm was measured using a Spark microplate reader (Tecan, Männedorf, Switzerland).

### 4.13. Statistical Evaluation

For the statistical evaluation of the functional assays, a Kruskal–Wallis test was performed in R. To correct for multiple comparisons, a Dunn’s multiple comparison test implemented in the Fisheries Stock Analysis (FSA), R Package Version 0.8.25; [76] was performed. Uncorrected *p*-values for the comparisons of interest were extracted, to avoid correction for comparisons that were not investigated. Uncorrected *p*-values were corrected for multiple testing using the FDR [71] implemented in the multtest package [72]. Each experimental assay was performed independently 4–7 times, with 4–8 technical replicates each.

## 5. Conclusions

In this study, a laser-induced CNV mouse model was used to identify robustly dysregulated miRNAs in blood and RPE/choroidal tissue. Each of these miRNAs, namely mmu-mir-486a-5p and mmu-mir-92a-3p, has previously been linked to pathologic processes related to AMD pathogenesis.

## Supplementary Materials

Supplementary materials can be found at https://www.mdpi.com/1422-0067/21/8/2689/s1.

## Abbreviations

AMD	age-related macular degeneration
ARPE-19	human retinal pigment epithelium cell line
BV-2	murine microglial cell line
C166	murine endothelial yolk sac cell line
cmiRNA	circulating microRNA
CNV	choroidal neovascularization
DPBS	Dulbecco’s phosphate buffered saline
E-PAP	*E. coli* Poly (A) Polymerase I
FCS	fetal calf serum
FDR	false discovery rate
HUVEC	human umbilical vein endothelial cells
LPS	lipopolysaccharide
miRNA	microRNA
nAMD	neovascular age-related macular degeneration
NGS	next generation sequencing
NO	nitric oxide
RPE	retinal pigment epithelium cells
RT-qPCR	quantitative reverse transcription PCR
SD-OCT	spectral domain optical coherence tomography
SE	standard error
TMM	trimmed mean of M-values
VEGF	vascular endothelial growth factor
