# Supplementary material for: A Circulating MicroRNA Profile in a Laser-Induced Mouse Model of Choroidal Neovascularization
Source: Int J Mol Sci. 2020 Apr 13;21(8):2689. doi: 10.3390/ijms21082689 (PMC7216141; doi:10.3390/ijms21082689)
Supplement: Supplementary file 1 [file ijms-21-02689-s001.pdf]

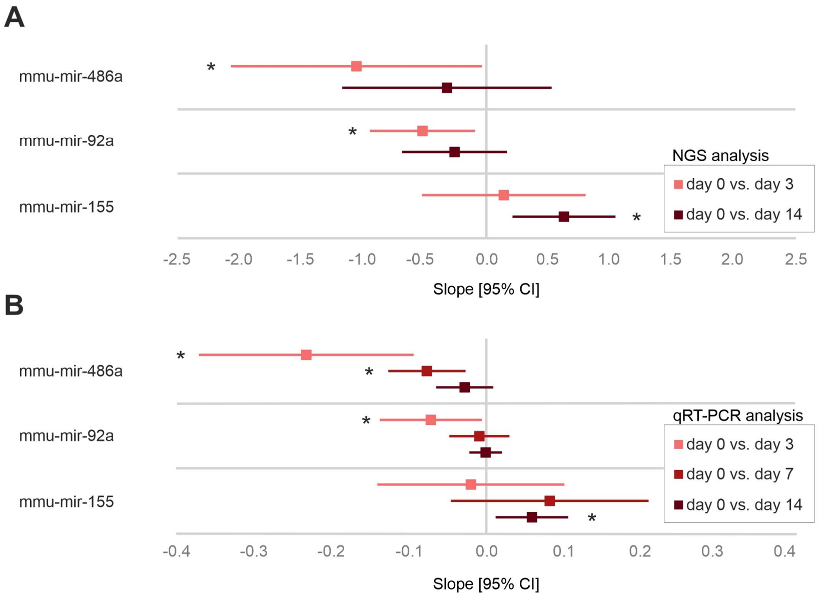

Supplementary Figure 1. CmiRNA regulation after laser-induced CNV in a discovery and a replication study. (a) The slope of three significantly dysregulated cmiRNAs in the discovery study (b) and replication study are shown. For the independent replication study using RT-qPCR, two additional batches of mice were treated with an argon laser, including 6 and 12 mice respectively. Shown are slopes with 95% confidence intervals for the three cmiRNAs for three different time points after laser treatment (days 3, 7 and 14) in comparison to day 0 as baseline control. All three cmiRNAs were differentially regulated at one or several time points of measurement. * p-value < 0.05 (linear regression model).

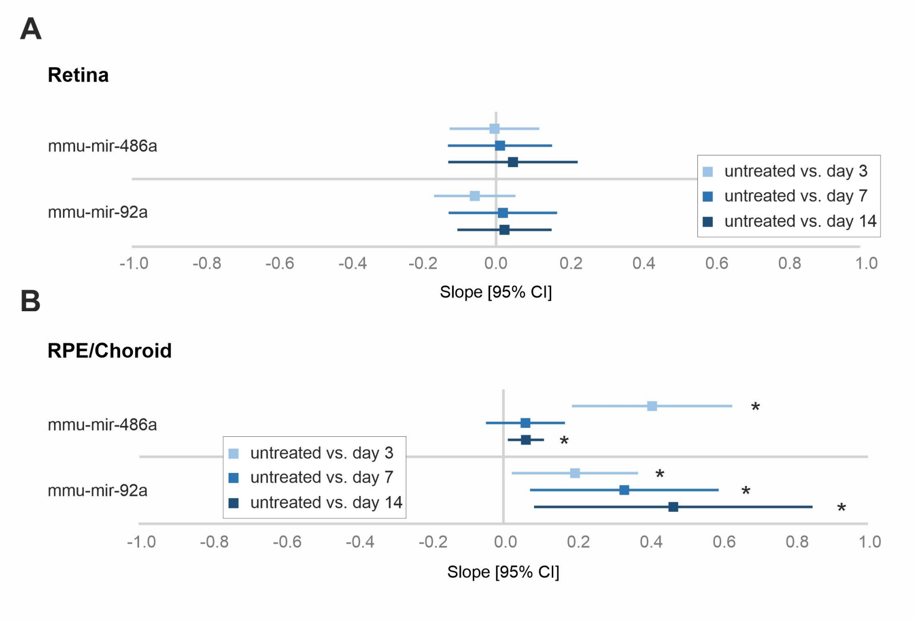

Supplementary Figure 2. MicroRNA regulation in ocular tissue after laser-induced CNV. (a) MiRNA expression levels of the two cmiRNAs dysregulated in blood samples, in retinal tissue and (b) RPE/choroidal tissue. Given are slopes with 95% confidence intervals which represent changes in expression of the respective miRNA expression in comparison to untreated control mice. * Q-value < 0.05 (linear regression model, FDR corrected).
